# P56 Improving the OPAT referral pathway for syphilis with neurological, ophthalmic or otic involvement: a service evaluation to inform pathway development

**DOI:** 10.1093/jacamr/dlag102.062

**Published:** 2026-06-26

**Authors:** Kieran Killington, Rajeni Thangarajah, Julia Bilinska, Carolyn Hemsley

**Affiliations:** Guy’s and St Thomas’ NHS Foundation Trust, London, UK; Imperial College London, London, UK; Guy’s and St Thomas’ NHS Foundation Trust, London, UK; Guy’s and St Thomas’ NHS Foundation Trust, London, UK; Guy’s and St Thomas’ NHS Foundation Trust, London, UK

## Abstract

**Background:**

Syphilis, caused by *Treponema pallidum,* may present with neurological, ophthalmic, or otological involvement. In line with British Association for Sexual Health and HIV guidelines,^1^ treatment options include intramuscular (IM) procaine penicillin, oral (PO) doxycycline or IV ceftriaxone, the latter supported via the outpatient parenteral antimicrobial therapy (OPAT) service at Guys’ and St Thomas’ NHS Foundation Trust (GSTT). Adjunctive steroids are recommended for 72 h, starting 24 h prior to antimicrobial therapy, to reduce the risk of Jarisch-Herxheimer (JH) reactions.

**Objectives:**

To review referrals for neurosyphilis, ocular syphilis or otosyphilis to the GSTT OPAT service, evaluate variation in current practice, and inform development of a standardized referral pathway and digital decision-support tool.

**Methods:**

We conducted a retrospective service evaluation of patients identified between 1 January and 31 December 2025 using electronic records and an internal OPAT database. Cases were identified through diagnostic coding for neurosyphilis, ocular syphilis, and otosyphilis, supplemented by searches for procaine penicillin and prednisolone prescribing within the sexual health service. Data collected included demographics, diagnosis, investigations, treatment modality, OPAT referral content, corticosteroid prescribing, time to treatment, and follow-up documentation.

**Results:**

Twenty-eight patients with suspected or confirmed neurosyphilis, ocular syphilis or otosyphilis were identified (median age 43 years, range 25 – 82 years; 25 male, 3 female). Sexual orientation was recorded for 21 patients - 13 gay, 7 heterosexual and 1 bisexual. Diagnoses included neurosyphilis (*n*=20), ocular syphilis (*n*=5), neurosyphilis with ophthalmic involvement (*n*=2) and otosyphilis (*n*=1). Lumbar puncture was performed in 3 suspected neurosyphilis cases, where 2 demonstrated cerebrospinal fluid findings indicative of infection. Among 7 patients with suspected ocular involvement, 6 were reviewed by ophthalmology and anterior uveitis confirmed in 5 patients. Eighteen of 28 patients (64%) were managed without OPAT (IM procaine penicillin, *n*=9; PO doxycycline, *n*=9). Treatment was documented as patient-preference driven in 11 cases, with rationale unclear in the remainder. Ten patients (36%) were referred to OPAT for IV ceftriaxone. Corticosteroids were prescribed in line with recommended timing in 7 cases, with variation in documentation or use in the remainder. Corticosteroids were contraindicated in 1. Mean time from OPAT acceptance to treatment initiation was 4 days (range 0–8). Follow-up documentation varied; where recorded, most patients demonstrated clinical and/or serological improvement.

**Conclusions:**

This evaluation demonstrates variation in documentation of treatment rationale, steroid timing and follow-up. These findings informed development of a standardized referral pathway incorporating an electronic referral template, clearer guidance on corticosteroid initiation and defined 3 month follow-up. This approach supports consistent, guideline-aligned care and optimizes antimicrobial stewardship across care settings. Our model highlights the value of standardized, digitally supported referral processes for managing complex sexually transmitted infections through OPAT services and provides a scalable approach for other centres.
